# Effects of extreme acceleration, microgravity, and deceleration on *Bacillus subtilis* onboard a suborbital space flight

**DOI:** 10.1038/s41526-025-00526-4

**Published:** 2025-10-06

**Authors:** Eric Yang, Jibin Jeffrey Dhanaraj, Palalle G. Tharushi Perera, Zoltan Vilagosh, Gail N. Iles, Stefan Krämer, Gunnar Florin, Denver P. Linklater, The Hong Phong Nguyen, Chaitali Dekiwadia, Saulius Juodkazis, Rodney J. Croft, Elena P. Ivanova

**Affiliations:** 1 ResearchSat Pty, Innovation and Collaboration Centre, University of South Australia, Adelaide, SA Australia; 2 School of Science, STEM College, RMIT University, Melbourne, VIC Australia; 3 Swedish Space Corporation, Solna, Sweden; 4 RMIT Microscopy & MicroAnalysis Facility, STEM College, RMIT University, Melbourne, VIC Australia; 5 Optical Sciences Centre, Swinburne University of Technology, Hawthorn, VIC Australia; 6 School of Psychology, Australian Centre for Electromagnetic Bioeffects Research, University of Wollongong, Wollongong, NSW Australia

**Keywords:** Microbiology, Biogeochemistry

## Abstract

The effects of extreme acceleration, microgravity, and deceleration of a suborbital spaceflight on *Bacillus subtilis* ATCC 6051 spores were investigated. *B. subtilis* spores were exposed to a maximum acceleration of ~13 g, a microgravity phase of 6 min, deceleration of 30 g (~300 m/s^2^), and a maximum rotating velocity of 220 ^o^s^−1^ upon re-entry into Earth’s atmosphere. Post-flight analysis showed the spores exhibited no change in morphology or viability, confirming that *B. subtilis* spores are resilient to space flight conditions.

## Body

Humans began travelling into space in 1961 with single orbits of the Earth. With the maturity of launch vehicles and life-supporting systems, this progressed to long-term stays of days and weeks onboard space shuttles and early orbiting laboratories^1^. Since the year 2000, there has been a continuous human presence in space onboard the International Space Station (ISS). While typical ISS missions were planned for up to six months, astronauts have also remained in space longer due to mission requirements^1^. To date, over 650 people have flown into space, with rocket launch being the only method of access. A growing area of interest for human spaceflight is space tourism which utilizes suborbital flights of a few minutes in duration. As humans go into space, so do the trillions of microbial cells harboured in the gut, on the skin, and in the respiratory and genital epithelium that make up the human microbiota. These flights have the potential to induce changes in bacterial behaviour triggered by altered gravity sequences, providing an interesting field for further exploration. With the recent increase in commercial space opportunities, it is expected that more humans, as well as their microbiota, will be subjected to altered gravity states following a sequence of hypo-gravity, microgravity, and hyper-gravity conditions.

Microorganisms have been studied in space for many decades, both inside spacecraft, and upon exposure to the harsh conditions of space. With launch conditions considered, such microbes would have experienced extreme conditions such as solar and galactic radiation, and microgravity changes^2–4^. However, most studies focus only on the behaviour of microbes over the long term (a matter of weeks) in the microgravity environment. For example, analysis of transcriptome profiles of *B. subtilis* after two separate space missions indicated that 91 genes were differentially expressed in comparison to the ground controls^5^. In bacteria exposed to space conditions, there was a significant increase in the expression of genes related to biofilm formation, biotin and arginine biosynthesis, siderophore production, manganese transport, toxin production and resistance, and the inhibition of sporulation. It was hypothesized that the oxygen availability between ground, onboard and cell sedimentation might be the reason for the changes in genetic profiling^6^.

There is currently limited information on the effects resulting from rapidly altered gravity sequences lasting in the timescale of minutes. The effects of specific suborbital conditions on microbes, spores, and bacteria are therefore largely unexplored and remain an area to be further investigated. Spores of *B. subtilis* are known to be highly resistant to the harsh conditions seen beyond the Earth’s atmosphere, therefore they are presently being used as indicators for planetary protection^6–8^. *B. subtilis* is a bacterial species capable of sustained growth under a wide range of environmental conditions. When exposed to unfavourable environments, *B*. *subtilis* survives through the formation of spores, a process that is characteristically triggered by a shortage of nutrients and stress^6,9,10^. Once a spore is formed, it can survive severe environmental conditions such as exposure to extreme heat, hazardous chemicals, and ionising radiation^11–14^. Given that *B. subtilis* spores are comprehensively characterized, they were chosen as the ideal candidate for initial studies on the effect of suborbital flight conditions on microbial life^13,15^. As a pilot study to investigate the impact of rapid changes in acceleration, microgravity conditions, and deceleration in the order of minutes, this research aims to investigate the effect of suborbital flight conditions on *B. subtilis* ATCC 6051 spores under a controlled environment of constant temperature and pressure.

## Suborbital launch vehicle

In mid-2022, ResearchSat Ltd collaborated with RMIT University to undertake fundamental research on biological specimens in microgravity aboard a suborbital flight scheduled for November 2022. For the same flight, ResearchSat was contracted by Numedico Technologies, an industrial partner specializing in drug delivery, to develop a drug microencapsulation payload for double emulsion Water-in-Oil-in-Water (W/O/W) research.

**Fig. 1:**
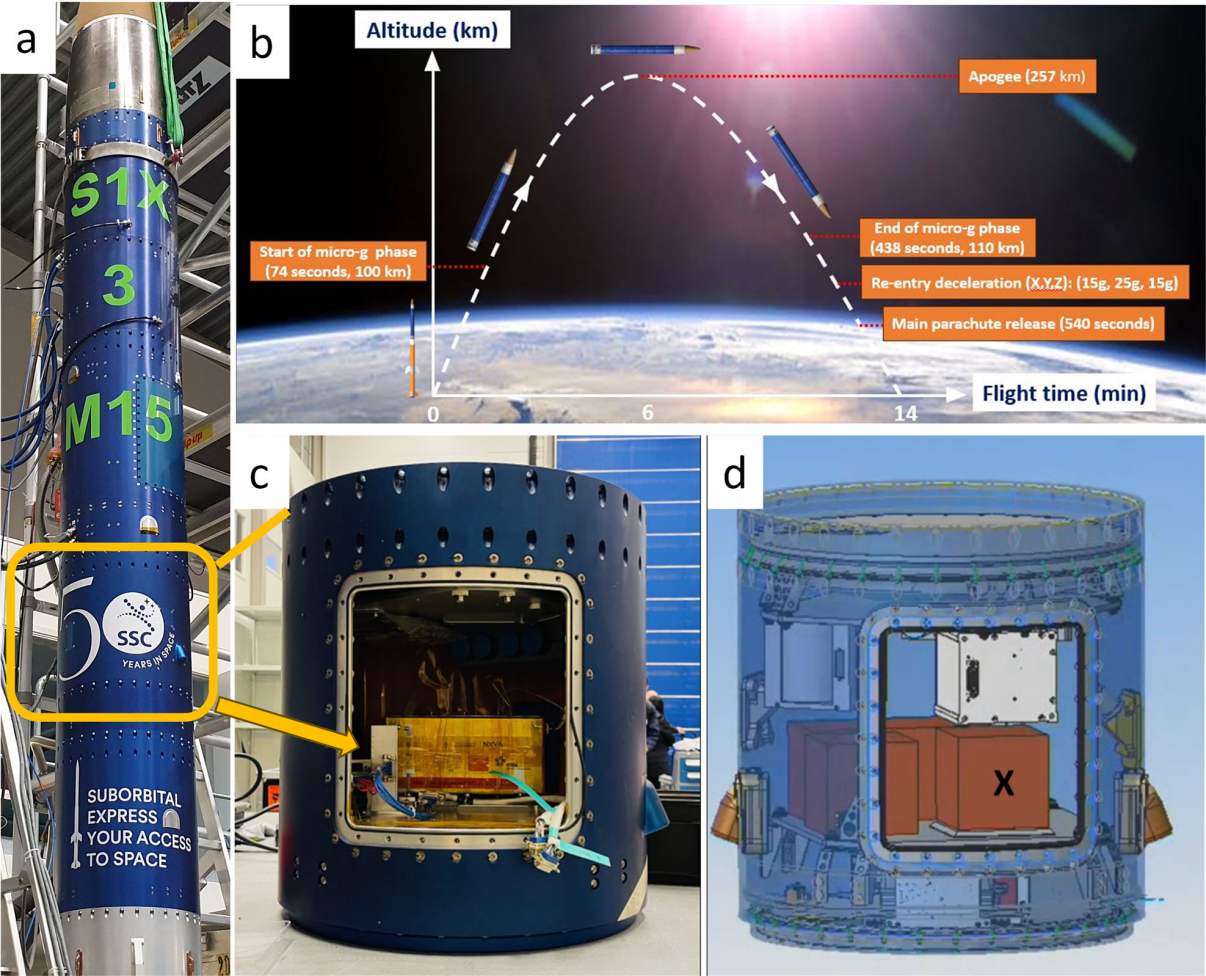
Rocket flight stages and payload location. **a** The payload section of the Suborbital Express 3 - M15 sounding rocket on the assembly pad. **b** Flight trajectory of Suborbital Express 3 - M15 presenting major flight events at relevant altitudes and timings. **c** Rocket section for rideshare payloads. **d** CAD drawing indicating the location of ADI-α payload inside the rideshare module.

Integration of the Advanced Diagnostic Laboratory-α or in short, ADI-α payload was conducted by securing the bottom surface of the payload inside of the rocket segment dedicated for payload ‘rideshare’ (Fig. 1). This particular rocket segment can host up to six payloads of 1U (10 × 10 × 10 cm) or 2U (10 × 10 × 20 cm) form factor, in which the ADI-α payload shares its internal volume with payloads of other institutions at the time of launch. The two-stage SubOrbital Express 3 - M15 rocket (S1X-3 M15), largely constructed of aluminium, features a heat shielding protection to separate the internal payloads from the extreme heat induced during rocket re-entry into the atmosphere and a parachute system for safe recovery of the payloads.

## Flight conditions

**Fig. 2:**
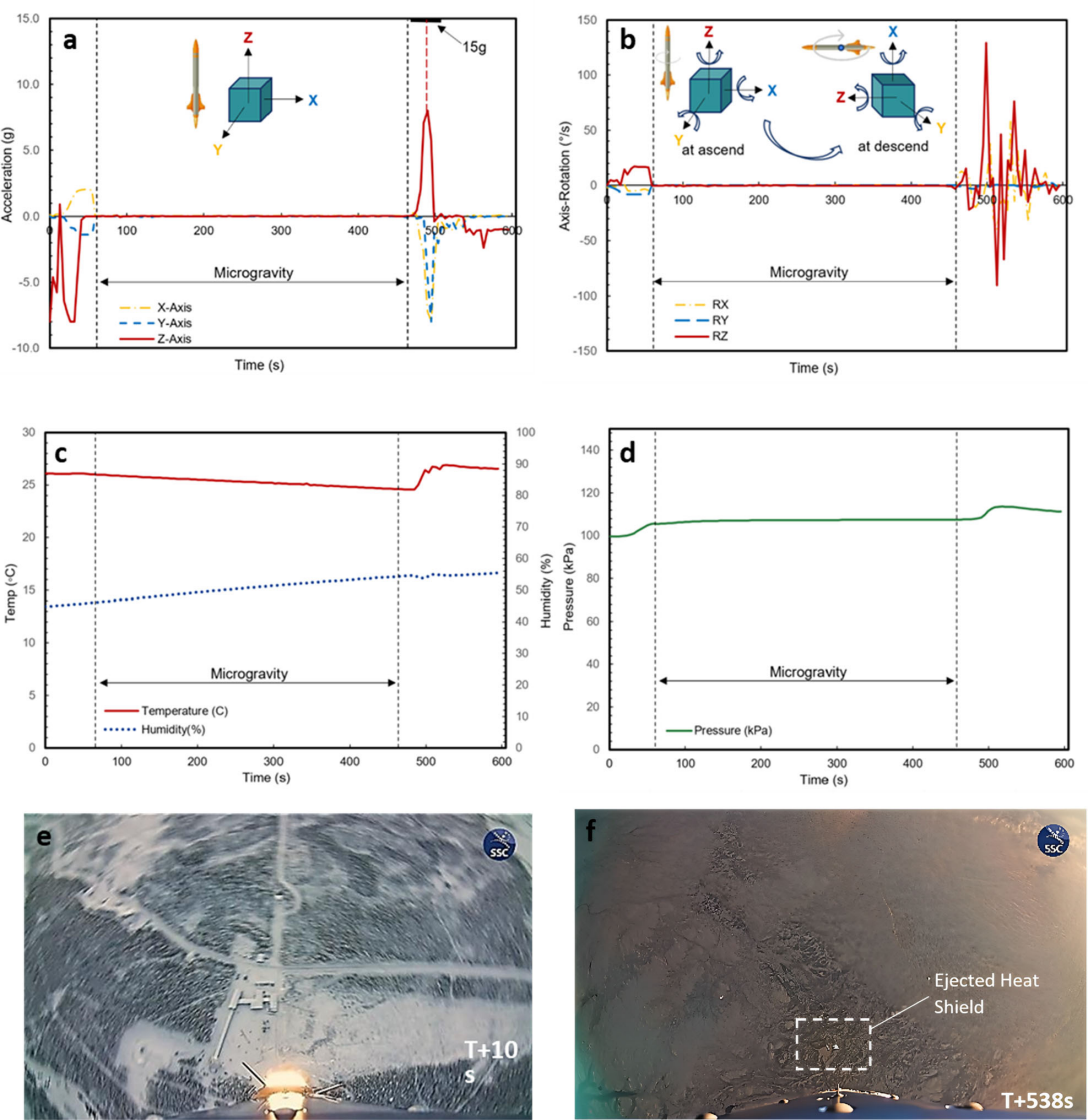
Flight condition data captured via instrumentation. **a** ADI-α Acceleration. **b** ADI-α Angular-velocity. **c** ADI-α temperature and humidity. **d** ADI-α pressure. **e** Induced spin captured by S1X-3 M15 external camera. **f** Heat shield ejection captured by S1X-3 M15 external camera.

Suborbital Express 3 - M15 Rocket launched successfully on 23-Nov-2022 09:23 am local time from Esrange Space Centre, Northern Sweden, according to ADI-α Payload flight data. A maximum acceleration of 13 g (~147 m/s^2^) was achieved during the second stage burn phase (see acceleration curve Fig. 2). 9 g (during the 1st stage burn phase) was recorded at lift-off (T + 0 s), with an immediate axial-spin induced in the z axis to enhance flight stability (Fig. 2a, e). At the main engine cut-off (T + 40 s), a mechanical yo-yo device was deployed shortly after to immediately cease the spin. This is indicated by the abrupt drop of z-acceleration to 0 g in Fig. 2a, coinciding with the transition into space (as defined by the von Kármán line) at 100 km altitude. With motor separation occurring after the anti-spin device is deployed (T + 58 s), the remaining rocket segment proceeded with the flight following an un-powered parabolic trajectory with the nose pointing upwards, which recorded ~418 s of flight above von Kármán line before payload re-entry (T + 468 s).

On the descent, the cold gas rate control system was activated to transition the rocket from the “nose-up” posture to a “flat-spinning” posture upon re-entry, as presented in Fig. 2b. The transition helps to reduce the descent velocity of the rocket to appropriate conditions for the ejection of the heat shield presented in Fig. 2f and enables the parachute to be deployed at its target altitude. ADI-α recorded a brief but substantial z-axis deceleration above 13 g during T + 480 s ~ 500 s per Fig. 2a. With the data capture range of ADI-α accelerometer limited to below 9 g, it is believed that the peak deceleration data recorded during re-entry is significantly below the actual value. In addition, ADI-α data also recorded violent spinning of the payload in all three axes (Fig. 2b). The payload continued to descend with the rocket segment over the following 2 min, and eventually concluded upon touchdown at T + 605 s.

During the duration of the flight, the temperature and humidity of the payload were also maintained at standard room conditions throughout the flight, with a slight increase in temperature to 28 °C, and humidity to 55% upon payload re-entry (Fig. 2c). The ADI-α payload was always pressurized and maintained at ambient pressure 100 ± 10 kPa (absolute) (Fig. 2d). The Sub-Orbital space flight of S1X-3 M15 reached a maximum altitude of 257 km at T + 255 s, which facilitates a total microgravity duration of 363 s. A summary of flight parameters is provided in Table 1, with a detailed evaluation of acceleration, rotation speed, and environmental parameters data captured by ADI-α presented in Fig. 2a–d.

**Table 1:** Suborbital express SX-3 flight parameters

Launch date	23 November 2022
Launch location	Esrange Space Centre, Kiruna, Sweden
Launch time	09:23 am local time (T + 0), which corresponds to 08:23 UTC
Start of microgravity	T + 75 s
End of <10^−4 ^g microgravity phase	T + 438 s
Total microgravity time	363 s
Heat shield ejection/drogue chute release	T + 537 s
Main parachute release	T + 540 s

## Assessment of spore viability and morphology

*B. subtilis* spores were subjected to a suborbital space flight, which involved rapidly altering gravity conditions. The spores experienced controlled exposure to rapid acceleration, microgravity, and subsequent deceleration over a short period (~10 min). Hence, we assessed the biological response of dormant spores followed by the flight. We refer to the spores exposed to suborbital flight conditions henceforth as ‘astrospores’ and the control group as ‘ground spores’.

**Fig. 3:**
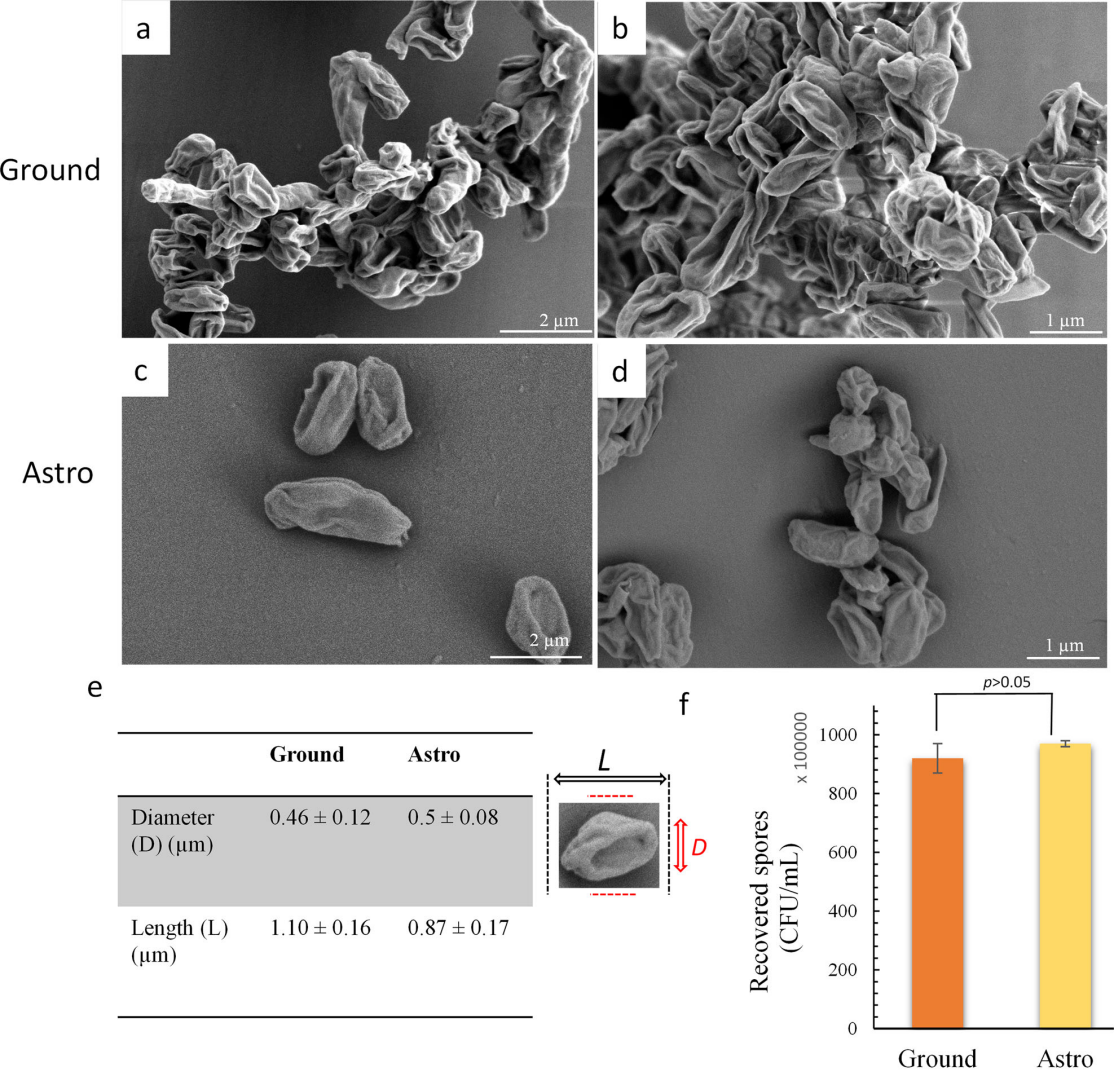
Morphological analysis of spores. Typical SEM images of freeze-dried spores: **a**, **b** Ground spores. **c**, **d** Astrospores, post space flight. The morphology of the astro spores appear to be like that of the ground sample. **e** The spore dimensions were estimated using SEM micrographs. The inset of an individual spore represents the spores’ dimensions. No statistically significant differences were recorded between the two groups. **f** The spores’ germination (nutrient agar, Oxoid); ~9.7 ×10^7^ CFU/mL bacterial cells were recovered. Statistical analysis of recovered bacterial cells from control group and after the space flight failed to detect any changes relative to the ground control.

Following the suborbital space flight, the spores’ viability and morphology were comparatively analysed using the direct plate counting technique and SEM imaging (Fig. 3a–d). No changes in viability were recorded between the post flight astrospores and the ground spores. The number of viable astrospores was recorded to be 9.7 ×10^7^ CFU/mL, whereas the number of viable ground spores was 9.2 ×10^7^ CFU/mL; there were no statistically significant differences recorded between the experimental groups, *t(*9*)* = 9*, p* = 0.07.

The effect on spore morphology directly resulting from the flight conditions was further investigated via comparative analysis of the spores’ dimensions, i.e., length (*L*) and diameter (D*)*. The *L* recorded among the two experimental groups did not exhibit statistically significant differences, *t*(19) = 2.44, *p* = 0.13. The astrospores exhibited a typical morphology when compared to that of the ground spores (Fig. 3). Astrospores maintained the original structure of the surface coats (Fig. 3). The quantification of the spore diameter (d) revealed a spore diameter in the range of 0.9–1.1 µm. The astrospores had a diameter of 0.9 µm ± 0.2 µm, whereas the ground spores had a diameter of 1.1 µm ± 0.2 µm. There were no statistically significant differences among the astrospores and the ground spore group, *t*(19) = 0.22, *p* = 0.84.

Thus, the results obtained in this study indicated that *B. subtilis* spores were able to withstand suborbital flight conditions (fast and extreme acceleration, microgravity, and deceleration), and contribute toward further understanding the extent of the physiological resilience of *B. subtilis* spores. It was previously reported that *B. subtilis* survived under long-term microgravity conditions in laboratory experiments^12^. It was also shown that *B. subtilis* spores can not only endure extremely high acceleration of over 400,000 × *g* for up to 60 h^13^ but have survived nearly 6 years of space microgravity conditions^3^. The extraordinary resistance of the bacterial spore is thought to be due to its multiple features, including spore coat, which consists of ~70 different proteins that protect the spore from environmental insults^9,16–18^.

Bacteria travel wherever humans go. If bacteria can endure rapid changes in acceleration or microgravity, then it is important to understand such changes, particularly given that suborbital space flight will become more common in the future. In this study, we investigated the morphology and viability of spores post-suborbital flight inside the ADI-αpayload compared to spores maintained on the ground. The effects of flight conditions endured onboard the SubOrbital Express 3 - M15 sounding rocket by *B. subtilis* spores have not been studied previously; this includes the extreme initial acceleration of only 74.7 s, at which point the payload was at a height of 100 km, and the maximum acceleration on take-off was above 13 g. Remarkably, following exposure to such fast and extreme acceleration, microgravity, and deceleration episodes, the astrospores did not exhibit any changes in morphology or viability in comparison to the ground controls. Our results confirmed that the dry spores are resistant to extreme environmental conditions:indeed, Kennedy et al. reported that dry spores were able to survive for more than 500 years^19^. The potential for changes in cell behaviour triggered by ASMD episodes needs to be further explored by obtaining relevant data on de-coated and well hydrated spores that are less resistant to heat and chemical, and studying biological adaptations, allowing researchers and pharmaceutical companies to take advantage of these adaptations to conduct innovative life science experiments.

## Methods

### Advanced diagnostic laboratory-α payload

ADI-α Payload, is a suborbital scientific payload developed by ResearchSat for the suborbital mission Suborbital Express 3 - M15 (Fig. 1a). Within ADI-α, two individual experiment chambers were developed to accommodate the microbial experiment for RMIT, a yeast cell culture experiment by ResearchSat for internal research as well as the drug microencapsulation experiment for Numedico. The Payload bus is made from aluminium 6061. The structural design of the Payload consists of an L-shaped cover panel and a body structure interfaced with an internal chassis attached to the body utilizing screws. The experiments are housed individually in experimental chambers known as Lab Modules. The Lab Modules are identical, and each contains a suite of electronics, sensors, and microfluidic chips to autonomously run the scientific experiments.

Power is supplied from the Launch vehicle 6 h before lift-off (LO). At 10 minutes before launch, the flight computer is powered up and the payload is autonomously put into safe mode. At vehicle lift-off, the LO signal sent to the flight computer initiates all sensory systems and storage media, enabling onboard sensors to begin logging flight data, while all onboard cameras, fluid pumps, and spectrometers will remain dormant in standby mode. Upon receiving the microgravity signal (μG), all systems will be turned on immediately to begin conducting and recording the experiments. All payload systems will function until final power cut-off, expected on parachuted Landing as the final stage of the return flight.

The ADI-α payload was integrated onto the sounding rocket (Fig. 1c, d), for the Suborbital Express 3 - M15 launch campaign hosted by the Swedish Space Corporation (SSC)^15,20^. The SSC S1X-3 M15 campaign features a two-stage sounding rocket powered by a VSB-30 engine combination, consisting of five payload modules of 43 cm in diameter and up to 110 cm in height. A typical flight sequence including de-spin and re-entry time events is included in reference^21^. After the engine was jettisoned, the payload bay including all payload modules remain intact throughout the flight and returned to earth via parachute for soft touch-down. The main flight events and positions of the rocket in space at each stage is illustrated in Fig. 1b. Both the rocket motors and payload modules were retrieved post-flight, with no launch or space debris generated.

### RMIT experimental module

**Fig. 4:**
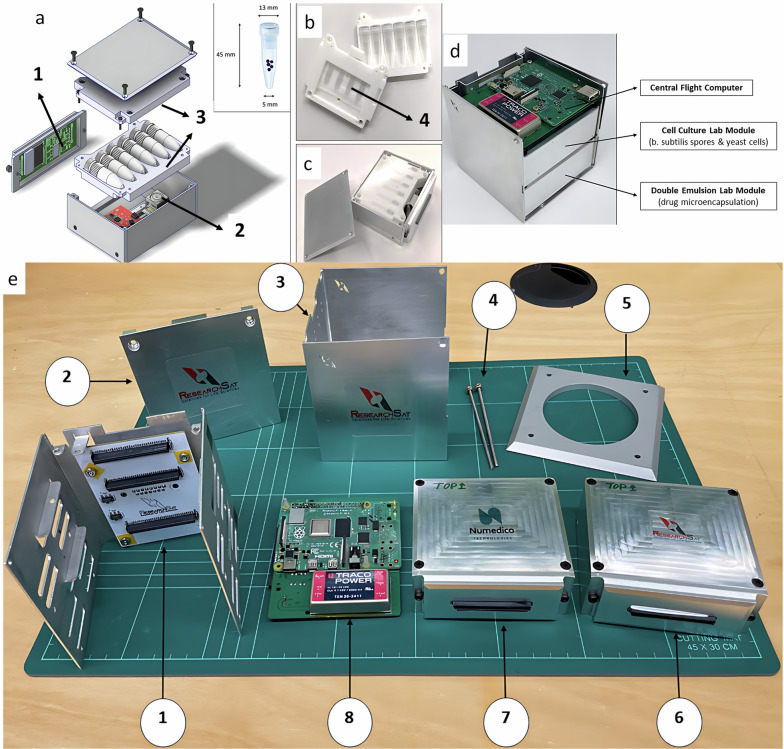
ADI- α payload. **a** Cell culture lab module. **a** 1. CC Module microcontroller & sensor suite 2. Spectral sensor and x-y motion microscopic camera 3, Microtube used for the *B. subtilis* spores (inset). **b** Microtube holder. **c** Cell culture lab module after assembly. **d** ADI-α with some of the major payload subsystems: 1) Chassis 2) Case cover 3) Case box 4) Lock pins 5) Payload Interface plate 6) Cell Culture Lab Module 7) Double Emulsion Lab Module 8) Central flight computer. **e** The flight computer and the chamber containing two lab modules.

To facilitate this research, a custom designed microtube holder was jointly developed by RST and RMIT University. The microtube holder was fabricated using additive manufacturing (Fig. 4a–c). The freeze-dried *B. subtilis* spores (1 mg, prepared as described in section 2.5), were placed in equal amount into six microtubes. The microtubes were then secured inside the ADI-α payload (Fig. 4d) with the microtube holder and securely fixed inside Lab Module.

1. The microtube holder was designed and fabricated via additive manufacturing and is made from commercially sourced PLA material.

### Integration & testing

The ADI-α payload for participating in the Payload Integration Tests was transported to Solna, Stockholm, and passed a series of certification tests which included:

- Payload mechanical integration
- Interface verification
- Induced vibration
- Payload electrical verification
- Payload functional test
- Payload Electro-magnetic interference check-out
- Flight simulation

The ADI-α payload achieved certification for flight readiness on the 28^th^ of September 2022. Following this certification, the payload along with all the other scientific experiments scheduled for the suborbital mission were sent to Esrange Space Center in Kiruna, about 1250 km road distance north of Solna, located at 68 °N latitude, within the Arctic circle (66 °N).

As the ADI-α payload was planned to carry biological samples on the suborbital flight which was scheduled to take place two months post integration testing, it was determined that the biological samples would be sent closer to the launch date rather than for the integration test phase.

### Growth of *B. subtilis* and spore sample preparation

*B. subtilis* ATCC 6051 were purchased from the American Type Culture Collection (ATCC, Manassas, VA, USA), and were grown on nutrient agar (NA, Oxoid, Basingstoke, England) for one week to induce sporulation, as described elsewhere^22^. The collection of spores was achieved by washing them in 2 mL of sterile H_2_O. The spore suspension was centrifuged at 4500 rpm for 10 min and washed twice with sterile cold H_2_O. The final debris free spore pellet was resuspended in 2 mL of sterile H_2_O and exposed to 80 °C for 20 min to kill all the vegetative cells followed by brief sonication. A spore staining, followed by microscopic observation, was carried out to confirm the presence and purity (>95%) of the spores.

After a serial dilution, the number of viable spores were quantified using a direct plate counting technique^22^. Spores were exposed to freeze drying at −80 °C using a Freeze Dryer Configurator manufactured by Labconco Corporation, Kansas, United States. An aliquot of 10 µL of the spore suspension was distributed into 20 microtubes (Polypropylene (PP) 1.5 mL, (Sarstedt, Germany) and freeze dried. The initial concentration of spores per aliquot were in the range of 9.2 ×10^7^ ± 0.5 CFU/mL. The freeze-dried spore samples (six) were then transported via an air-tight case to the rocket launch site for integration. The remainder of the freeze-dried spores were kept on the ground at room temperature and used as the ‘ground’ control set. In addition, there were 6 negative controls that were also at room temperature.

### Scanning electron microscopy

The FEI Quanta FE SEM) scanning electron microscope (SEM) with a primary beam energy of 3 kV was used for imaging the samples post-flight. The dried spores were placed on carbon tape, the eppendorfs were cut open in a manner without disrupting the sample, and silver paint was applied onto the eppendorf base to prevent charging of the plastic while imaging. Prior to imaging, fixed spores were subjected to iridium (IR) sputtering (6-nm thick) using a Leica ACE600 Sputter coater (Vienna, Austria). The same procedure was applied to the control experimental groups. Ten SEM images with different magnifications were captured and analysed.

### Assessment of spore dimensions

The quantification of the spores’ dimensions including length (*L*) and diameter (*D*) was carried out using the Image J software (Image J plugin; National Institute of Health, Bethesda, MD, USA) by manually tracing the diameter (Fig. 4a; inset) of spores in ten different fields of view.

### Spore viability

Spore viability was tested using the direct plate counting technique. In brief, spore samples retrieved from space travel were mixed with 500 µL of sterile milliQ water to create a 1:50 dilution suspension. A series of dilutions were then performed using the same method, until four dilutions were created. After the dilution series, 30 µL from each dilution were plated onto nutrient agar (NA, Oxoid, Basingstoke, England) and incubated at 37 °C overnight with the appropriate controls. Colony forming units (CFUs) were counted after 24 h incubation according to the Eq. (1) listed below:

1 $${\rm{CFU}}=\frac{{\rm{colony\; count}}\times {\rm{dilution\; factor}}}{{\rm{volume\; plated}}({\rm{mL}})}$$

The CFU/mL was then expressed for the different experimental groups.

### Statistical analysis

Statistical data processing was conducted using the Statistical Package for the Social Sciences, SPSS 24.0 (SPSS, Chicago, IL, USA). Statistically significant differences (*p* < 0.05) among the experimental groups (astro and ground control) were calculated using an independent groups *t*-test, where the independent variables were the astro and ground control.

## Data Availability

The authors confirm that the data supporting the findings of this study are available within the article. Raw data generated during this study are available from the first author and/ or corresponding author on request.
